# A novel model of an identified Drosophila crawl motoneuron for investigating functional effects of ion channel type across larval developmental stages

**DOI:** 10.1186/1471-2202-12-S1-P258

**Published:** 2011-07-18

**Authors:** Cengiz Günay, Logesh Dharmar, Fred Sieling, Richard Baines, Astrid A Prinz

**Affiliations:** 1 Dept. Biology, Emory University, Atlanta, Georgia 30322, USA; 2 Biomedical Engineering Dept., Georgia Inst. Tech. And Emory Univ., Atlanta, Georgia, USA; 3 Life Sciences, University of Manchester, Manchester M13 9PT, UK

## 

Drosophila is a powerful genetic model system for investigating neuronal function. Most of the important membrane ion channel genes, such as voltage-gated sodium and potassium channels, were first identified and isolated in the fruit fly. Technical advances have now made possible direct electrophysiological recording of channels in central neurons, allowing the genetic advantages of this system to be applied to analysis of cellular and circuit function and homeostasis.

An important open question is the functional effect of channel splice variants that has recently been found in *Drosophila* neurons. Because of the experimental difficulties in the isolated expression of these splice variants, computational modeling becomes essential. In this work, we aim at assessing effects of Na channel splice variants [1] on neuronal activity by first building a full model neuron.

For this full model neuron, parameters for individual ion channels are required. Previous literature on Drosophila ion channel parameters are highly variable. Taking an average of such disparate neuronal parameter values have been shown to be unideal [2]. Furthermore in our case, these data were collected from different neuronal types and preparations. We are thus focusing on identified larval aCC and RP2 abdominal dorsomedial motoneurons, which innervate the dorsal muscles [3], for building a novel full motoneuron model. We obtain their channel parameters by fitting models to experimental voltage-clamp data.

We first present a minimal, isopotential spiking model neuron with transient and persistent sodium, delayed-rectifier, and A-type potassium channels. This model allows us to investigate the contributions of the two major types of A-type currents *Shal* and *Shaker* that are different in spatial expression over the neuron and in terms of electrophysiological and activity-related characteristics. We show how the *Shal* channel properties change between the larval stages of 1^st^ and 3^rd^ instar, and we show the effect of this change on neuronal activity characteristics. We then aim to add the calcium channel and potassium channels that are dependent on it. Overall, this neuron model will enable us to investigate the effect Na channel splice variants by varying half-activation and inactivation voltages and ratio of a persistent component.
